# Evaluation of the inhibitory effects of chloroform on *ortho*-chlorophenol- and chloroethene-dechlorinating *Desulfitobacterium* strains

**DOI:** 10.1186/2191-0855-3-30

**Published:** 2013-05-27

**Authors:** Taiki Futagami, Yuko Fukaki, Hidehiko Fujihara, Kaoru Takegawa, Masatoshi Goto, Kensuke Furukawa

**Affiliations:** 1 Department of Bioscience and Biotechnology, Kyushu University, 6-10-1 Hakozaki, Fukuoka, 812-8581, Japan; 2 Department of Food and Bioscience, Faculty of Food Science and Nutrition, Beppu University, Kitaishigaki 82, Beppu, Oita, 874-8501, Japan

**Keywords:** Desulfitobacterium, Reductive dechlorination, Organohalide respiration, Growth inhibition, Chloroform, Chlorophenol, Chloroethene

## Abstract

Organohalide-respiring *Desulfitobacterium* strains are believed to play an important role in the bioremediation and natural attenuation of chlorinated aliphatic and aromatic hydrocarbons. However, several studies have reported that chloroform significantly inhibits microbial reductive dechlorination of chloroethene. In this study, we examined the effect of chloroform on several *Desulfitobacterium* strains, including *ortho*-chlorophenol-dechlorinating *Desulfitobacterium dehalogenans* JW/IU-1 and *Desulfitobacterium hafniense* DCB-2, and also the chloroethene-dechlorinating strain *D. hafniense* TCE1. In medium containing 3-chloro-4-hydroxyphenylacetate as an electron acceptor, chloroform inhibited the growth of strains JW/IU-1 and DCB-2. Although chloroform did not directly inhibit dechlorination of 3-chloro-4-hydroxyphenylacetate by resting cells, cells cultivated with chloroform showed decreased dechlorination activity. Moreover, transcription of the gene encoding the reductive dehalogenase CprA decreased significantly in cells cultivated with chloroform. These results indicate that chloroform inhibits the growth and dechlorination activity of strains JW/IU-1 and DCB-2 via inhibition of *cprA* transcription. In contrast, cultivation of strain TCE1 in the presence of chloroform gave rise to a PceA reductive dehalogenase gene-deletion variant of strain TCE1; a similar phenomenon was observed in our previous study of chloroethene-dechlorinating *D. hafniense* strain Y51. Our results suggest that chloroform extensively inhibits the dechlorination activity of *Desulfitobacterium* strains, and that the inhibitory mechanism appears to differ between *ortho*-chlorophenol dechlorinators and chloroethene dechlorinators.

## Introduction

Organohalide respiration is an anaerobic process in which a halogenated organic compound serves as the electron acceptor. Various organohalide-respiring bacteria (OHRB) have been applied to the bioremediation of toxic chlorinated hydrocarbons in anaerobic environments (Smidt and de Vos 2004;Löffler and Edwards 2006;Maphosa et al. 2010). In this process, organohalides are reductively dehalogenated. For example, tetrachloroethene (PCE) is successively converted to trichloroethene (TCE), dichloroethene (*cis*-dichloroethene or *trans*-dichloroethene), vinyl chloride (VC), and finally nontoxic ethene.

Species of the genus *Desulfitobacterium* are some of the most frequently identified OHRB, and can utilize a variety of electron acceptors, such as nitrate, sulfite, fumarate, humic acids, and organohalides (Villemur et al. 2006). Most *Desulfitobacterium* isolates can respire with *ortho*-chlorophenol and/or chloroethene. For example, *Desulfitobacterium hafniense* strain DCB-2 and *Desulfitobacterium dehalogenans* strain JW/IU-1 respire with *ortho*-chlorophenols such as 3-chloro-4-hydroxyphenylacete (3-Cl-4-OHPA), whereas *D. hafniense* strains TCE1 and Y51 respire with PCE and TCE (Madsen and Licht 1992;Utkin et al. 1994;Gerritse et al. 1999;Suyama et al. 2001).

The dehalogenation reaction is catalyzed by reductive dehalogenase, which is the terminal reductase of the organohalide respiratory chain. The dehalogenation spectrum of each OHRB is believed to be determined by the type of this key enzyme. Both *D. dehalogenans* strain JW/IU-1 and *D. hafniense* strain DCB-2 possess *ortho*-chlorophenol reductive dehalogenase (CprA), for which the corresponding gene cluster (consisting of *cprA*, *cprB*, *cprC*, *cprD*, *cprE*, *cprK*, *cprT*, and *cprZ*) has been identified (Smidt et al. 2000;Christiansen et al. 1998;Kim et al. 2012). On the other hand, *D. hafniense* strains TCE1 and Y51 possess the PCE reductive dehalogenase (PceA) gene cluster, which consists of *pceA*, *pceB*, *pceC*, and *pceT* (Maillard et al. 2005;Suyama et al. 2002;Furukawa et al. 2005). The *pce* genes are surrounded by two copies of nearly identical insertion sequence (IS) elements that contain a gene homologous to the IS*256* family transposase; therefore, the *pce* genes can function as a typical composite transposon. In fact, several types of circular DNAs resulting from homologous recombination between two homologous IS elements and transposase-mediated excision and circularization have been identified in strains TCE1 and Y51 (Maillard et al. 2005;Futagami et al. 2006a;Duret et al. 2012).

The OHRB play a significant role in the detoxification of chlorinated hydrocarbons. However, chloroform (CF, trichloromethane), which is a common environmental pollutant that has both biotic and abiotic origins, reportedly inhibits microbial reductive dechlorination of chloroethene (Cappelletti et al. 2012). Bagley et al. (2000) showed that CF at a concentration of 4 μM completely inhibits degradation of PCE by a microcosm, while Duhamel et al. (2002) showed that a chloroethene-dechlorinating microcosm containing *Dehalococcoides* species is inhibited by 2.5 μM CF, resulting in accumulation of VC. Inhibitory effects of CF on the chloroethene dechlorination activity of the OHRB isolate *Dehalococcoides mccartyi* strain 195, and on the activity of purified PceA reductive dehalogenase from *Sulfurospirillum multivorans* have also been reported (Maymó-Gatell et al. 2001;Neumann et al. 1996;Löffler et al. 2013). In the case of *D. hafniense* strain Y51, we found that two different PCE-nondechlorinating variants become dominant in the presence of CF. One of these variants (designated the LD variant) lost all *pce* genes due to homologous recombination between the two IS elements. The other nondechlorinating variant lost one IS element located upstream of the *pce* gene cluster, in which a portion of the promoter region is located. This type of variant, designated as SD variant, cannot express *pce* genes. Both of these variants, but especially the LD variant, become dominant in the presence of CF, which inhibits the growth of wild type strain Y51 at concentrations as low as 1 μM, while both the LD and SD variants can grow normally at CF concentrations as high as 1 mM (Futagami et al. 2006b).

Although the effects of CF on the reductive dechlorination of chloroethene have been studied, how CF affects *ortho*-chlorophenol dechlorination remains to be elucidated. We therefore investigated the effect of CF on the growth, dechlorination activity, the genetic stability and transcription of the *cprA* gene in the *ortho*-chlorophenol-dechlorinating bacteria *D. dehalogenans* strain JW/IU-1 and *D. hafniense* strain DCB-2. In addition, we investigated the effect of CF on the chloroethene-dechlorinating bacterium *D. hafniense* strain TCE1, and compared the results to those from our previous study on *D. hafniense* strain Y51. We report here that CF strongly inhibits the dechlorination activity of *Desulfitobacterium* strains, and discuss the sigificance of the inhibitory effect of CF on *ortho*-chlorophenol-dechlorinating and chloroethene-dechlorinating *Desulfitobacterium* strains.

## Materials and methods

### Strains and cultivation

*Desulfitobacterium dehalogenans* strain JW/IU-1 (DSM 9161) and *D. hafniense* strains DCB-2 (DSM 10664) and TCE1 (DSM 12704) were obtained from the DSMZ microbe collection. *Desulfitobacterium hafniense* strain Y51 was previously isolated in our laboratory (Suyama et al. 2001). Strains JW/IU-1, DCB-2, TCE1, and Y51 were grown anaerobically at 30°C in MMYP medium (45.9 mM K_2_HPO_4_, 8.8 mM KH_2_PO_4_, 1.7 mM sodium citrate, 0.4 mM MgSO_4_ · 7H_2_O, yeast extract [2.0 g/L], 68.2 mM sodium pyruvate, and 4.0 μM resazurin sodium salt, pH 7.2) with 10 mM 3-Cl-4-OHPA or 5 mM sodium fumarate as an electron acceptor. CF was diluted in *N*,*N*-dimethylformamide and added to the culture medium to a final concentration of 1, 10, or 100 μM. Growth of all strains was assessed by optical density (OD) at 660 nm. Growth was assessed at least two times independently.

### Dechlorination of 3-Cl-4-OHPA by resting cells

Resting cells were prepared from cultures of strains JW/IU-1 and DCB2 grown in MMYP medium with 10 mM 3-Cl-4-OHPA and with or without 100 μM CF. The strains JW/IU-1 and DCB-2 were grown to late-log phase (OD_660_ = 0.2 and 0.4, respectively) and the cells were harvested by centrifugation at 4,000 × *g* for 10 min at 4°C. The cell pellet was washed with buffer (45.9 mM K_2_HPO_4_ and 8.8 mM KH_2_PO_4_, pH 7.5) and recentrifuged twice under the same conditions. The cells were then resuspended in phosphate buffer to an OD_660_ of 0.3. Dechlorination of 3-Cl-4-OHPA was measured in a 25-ml glass vial containing 5.0 ml of resting cell solution (OD_660_ = 0.25) containing 20 mM sodium pyruvate and 1.0 mM 3-Cl-4-OHPA. Each vial was sealed with a butyl rubber stopper and crimped. CF was added to a final concentration of 100 μM by syringe. All manipulations were performed under anaerobic conditions using centrifuge tubes with sealing caps (Nalgene, Rochester, NY) and an anaerobic grove box with an atmosphere of 85% N_2_, 5.0% CO_2_, and 10% H_2._ The reaction mixtures were incubated at 30°C with shaking at 120 rpm. The reaction solutions were collected with a syringe and filtered through a 0.2-μm PTFE filter (Millex-LG, Millipore, Bedford, MA). The 3-Cl-4-OHPA remaining in solution was quantified by high-performance liquid chromatography (HPLC) on a system equipped with a Waters 2487 UV detector operated at 280 nm (Waters, Milford, MA) and a YMC Pack Pro C18 RS column (YMC, Kyoto, Japan). The mobile phase was acetonitrile-water-acetic acid (30:70:1), and the column was eluted isocratically at a flow rate of 1 ml/min. Dechlorination activity was measured in triplicate samples and the results shown are the mean and standard deviation.

### Stability of the reductive dehalogenase genes

Strains JW/IU-1 and DCB-2 were cultivated in MMYP with 10 mM 3-Cl-4-OHPA and with or without 100 μM CF, and the cells were harvested at stationary phase. Genomic DNA was isolated according to established procedures (Wilson 1987), digested with *Eco*RI, subjected to agarose gel electrophoresis, and transferred onto a Biodyne B membrane (Pall Corporation, Pensacola, FL). Hybridization with digoxigenin (DIG)-labeled (Roche, Penzberg, Germany) DNA probes and detection by nitroblue tetrazolium/bromochloroindolylphosphate (NBT/BCIP) (Roche, Penzberg, Germany) were performed according to the manufacturer’s instructions. The *cprBA* gene probe for strain JW/IU-1 and *cprA* gene probe for strain DCB-2 were amplified using the DdehalogF1-DdehalogR1 and DCB2F1-DCB2-R1 primer sets, respectively (Table 1).

**Table 1:** Sequences of primers used in this study

**Primer name**	**Sequence (5′-3′)**	**Reference**
DdehalogF1	GCACTAATACTTGTGTATGTATTCC	This study
DdehalogR1	GACCACTGCAATGAGTG	This study
DdehalogF2	ATGAACCGCAGAAGCTTTCTG	This study
DdehalogR2	GGAACCAGGAATCTTCCTTG	This study
DCB2F1	CATGAACCGCAGAAGCTTTC	This study
DCB2R1	CAATGGTCTCCGTACCATAGC	This study

The sequences of GenBank accession numbers AF115542 and AY013365 were used for primer design.

Strains TCE1 and Y51 were cultivated in MMYP medium with 5 mM fumarate and with or without 100 μM CF and then harvested at stationary phase. Isolation of genomic DNA and Southern blot analysis were performed as described above. The DIG-labeled DNA probe used to detect the *pceA* gene was amplified as described previously (Futagami et al. 2006b).

### Northern blot analysis of *cprA* transcripts

Strains JW/IU-1 and DCB-2 were cultivated in MMYP medium with 10 mM 3-Cl-4-OHPA and with or without 100 μM CF and then harvested at the logarithmic phase. Total RNA was isolated from cells using RNAiso reagent (Takara, Shiga, Japan) according to the manufacturer’s protocol. The concentration of RNA was determined by measuring the absorbance at 260 nm with a spectrophotometer (UV-2550, Shimadzu, Kyoto, Japan). Total RNA (30 μg) was electrophoresed on a 1.0% agarose gel with 18% (vol/vol) formaldehyde and then transferred onto a Hybond N membrane (Amersham Biosciences, Buckinghamshire, UK). Hybridization with DIG-labeled *cprA* DNA probes and subsequent detection with NBT/BCIP were carried out according to the manufacturer’s instructions. The *cprA* gene probe for strain JW/IU-1 and the *cprA* gene probe for strain DCB-2 were amplified using the primer sets DdehalogF2-DdehalogR2 and DCB2F1-DCB2-R1, respectively (Table 1).

## Results

### Effect of CF on the growth of *D. dehalogenans* strain JW/IU-1 and *D. hafniense* strain DCB-2

**Figure 1:**
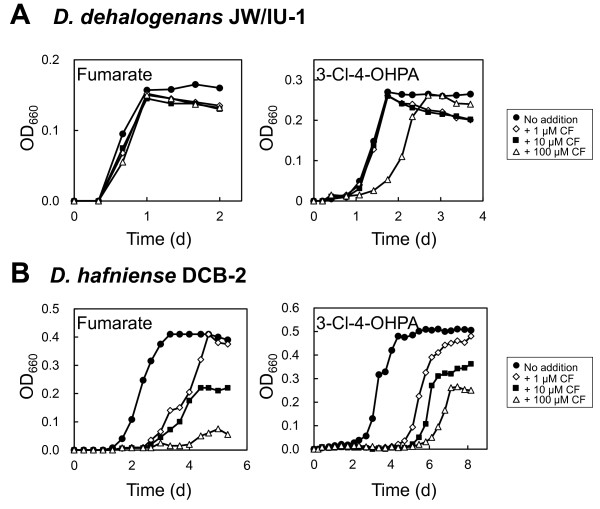
**Effect of chloroform on the growth of *ortho*-chlorophenol-dechlorinating strains *D. dehalogenans *JW/IU-1 (A) and *D. hafniense *DCB-2 (B).** Strains JW/IU-1 and DCB-2 were cultivated in medium containing fumarate or 3-Cl-4-OHPA alone (●) or supplemented with 1 μM CF (◇), 10 μM CF (■), or 100 μM CF (△). OD_660_, optical density at 660 nm.

To evaluate the inhibitory effect of CF on the growth of strains JW/IU-1 and DCB-2, we examined growth curves for cells cultured with and without CF at a concentration of 1, 10, and 100 μM in medium containing fumarate or 3-Cl-4-OHPA as the electron acceptor (Figure 1). In culture medium containing fumarate, the growth of strain JW/IU-1 was not inhibited by CF, even at a concentration of 100 μM, but in medium containing 3-Cl-4-OHPA, growth rate of strain JW/IU-1 was inhibited by 100 μM CF (Figure 1A). In contrast, CF caused extended lag phase and reduced cell yield of strain DCB-2 in a dose-dependent manner in the presence of fumarate or 3-Cl-4-OHPA (Figure 1B).

### Effect of CF on dechlorination of 3-Cl-4-OHPA by *D. dehalogenans* strain JW/IU-1 and *D. hafniense* strain DCB-2

**Figure 2:**
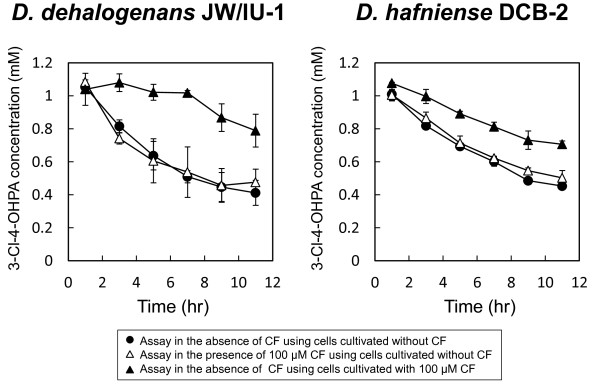
**Effect of chloroform on the reductive dechlorination activity of *D. dehalogenans *strain JW/IU-1 and *D. hafniense *strain DCB-2.** Strains JW/IU-1 and DCB-2 were cultivated in medium containing 3-Cl-4-OHPA, and resting cells were subjected to a dechlorination assay in the absence (●) of CF or presence (△) of 100 μM CF. Strains JW/IU-1 and DCB-2 were cultivated in medium containing 3-Cl-4-OHPA and 100 μM CF and the resting cells were subjected to the dechlorination assay (▲).

In medium containing 3-Cl-4-OHPA as the electron acceptor, strains JW/IU-1 and DCB-2 grew by organohalide respiration using 3-Cl-4-OHPA. Because 100 μM CF significantly inhibited the growth rate of strain JW/IU-1 and caused the extended lag phase and the reduced cell yield of strain DCB-2 in medium containing 3-Cl-4-OHPA (Figure 1), we investigated the effect of CF on dechlorination of 3-Cl-4-OHPA by resting cells prepared from strains JW/IU-1 and DCB-2 cultivated with or without 100 μM CF (Figure 2). The results of dechlorination assays showed that dechlorination of 3-Cl-4-OHPA by resting cells of strains JW/IU-1 and DCB-2 initially grown in the absence of CF was similar in both the presence and absence of 100 μM CF, indicating that 3-Cl-4-OHPA dehalogenase is not affected by CF. However, the dechlorination activity of resting cells of strains JW/IU-1 and DCB-2 initially grown in the presence of 100 μM CF was lower than that of resting cells cultivated without CF.

### Effect of CF on the stability and transcription of *cprA* in *D. dehalogenans* strain JW/IU-1 and *D. hafniense* strain DCB-2

**Figure 3:**
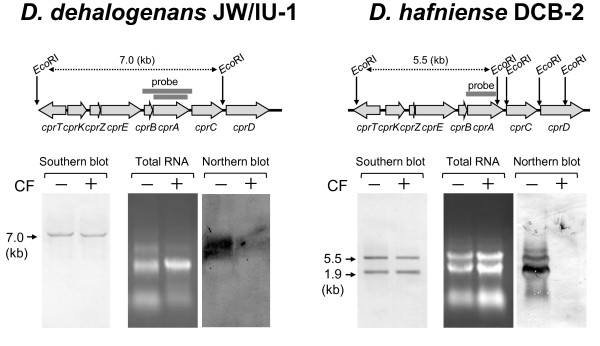
**Effect of chloroform on the stability and transcription of the *cprA *gene of *D. dehalogenans *JW/IU-1 and *D. hafniense *DCB-2.** The *cpr* gene clusters from *D. dehalogenans* IW/IU-DC1 and *D. hafniense* DCB-2 were derived from the sequences of GenBank accession number AF115542 and RefSeq accession number NC_011830, respectively.

Because strains JW/IU-1 and DCB-2 cultivated with 100 μM CF showed reduced dechlorination activity (Figure 2), we investigated the effect of CF on the stability and transcription of the *cprA* gene (Figure 3). Strains JW/IU-1 and DCB-2 cultivated in medium containing 3-Cl-4-OHPA with or without 100 μM CF were examined by Southern blot analysis. The *cprBA* and *cprA* genes were used as probes for strains JW/IU-1 and DCB-2, respectively. A fragment of DNA of ca. 7.0 kb from strain JW/IU-1 and fragments of ca. 5.5 and 1.9 kb from strain DCB-2 were detected, and the signal intensities measured for these fragments were the same for cells cultivated with or without CF (Figure 3). This result suggested that CF does not affect the structure of the *cpr* genes of strains JW/IU-1 and DCB-2.

In contrast, CF did affect the transcription of the *cprA* gene in strains JW/IU-1 and DCB-2 (Figure 3). The RNAs extracted from strains JW/IU-1 and DCB-2 cultivated in medium containing 3-Cl-4-OHPA and with or without 100 μM CF were subjected to northern blot analysis using *cprA* as a probe. The signal intensity of the *cprA* transcript from cells of strains JW/IU-1 and DCB-2 grown in the presence of CF was significantly lower than that of cells grown in the absence of CF. This result agreed with the reduction in 3-Cl-4-OHPA dechlorination activity of strains JW/IU-1 and DCB-2 when cells were grown in the presence of 100 μM CF (Figure 2).

### Effect of CF on the growth and *pceA*-stability of *D. hafniense* TCE1

**Figure 4:**
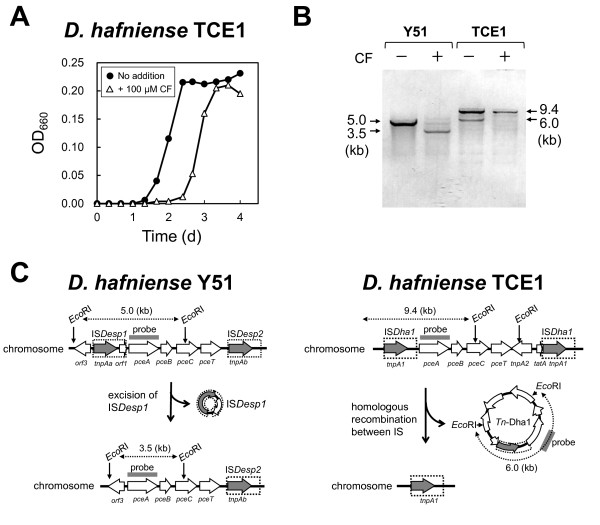
**Effect of CF on the growth (A) and stability of the *pceA *gene (B) of *D. hafniense *strain TCE1 in comparison with *D. hafniense *strain Y51, and genetic rearrangement of the *pce *gene cluster (C).** OD_660_, optical density at 660 nm (**A**). *Eco*RI-digested DNA (1 μg) was loaded into each lane of an agarose gel for Southern blot analysis (**B**). Representation of the *pce* gene clusters of strains TCE1 and Y51 were derived from the sequences of GenBank accession numbers AJ439608 and AY706985, respectively.

We also investigated the effect of CF on *D. hafniense* TCE1. This strain dechlorinates PCE and TCE and has a *pce* gene cluster similar to that of *D. hafniense* strain Y51 (Maillard et al. 2005;Futagami et al. 2006a). In the case of strain Y51, CF significantly inhibited the growth of wild type cells, but did not inhibit the growth of the PCE-nondechlorinating LD and SD variants missing the *pce* gene cluster or *pceA* promoter sequence, respectively (Futagami et al. 2006b). We first examined the effect of CF on the growth of strain TCE1 (Figure 4A). Cells of strain TCE1 were inoculated into medium with or without 100 μM CF and their growth was monitored. A growth lag of ca. 24 h was observed in the presence of CF, but the cells grew normally after this prolonged lag phase. This result was similar to that observed previously for *D. hafniense* Y51 (Futagami et al. 2006b).

Next, we examined the effect of CF on the stability of the *pce* gene cluster in *D. hafniense* TCE1, and compared the results to those obtained previously with *D. hafniense* Y51 (Figure 4B). Genomic DNA was extracted from cells cultivated with or without 100 μM CF and then subjected to Southern blot analysis using *pceA* as a probe. Southern blot analysis was used previously to detect the varieties of genetic alterations in *pce* gene clusters caused by IS elements in strains TCE1 and Y51 (Maillard et al., 2005;Futagami et al. 2006a;Futagami et al. 2006b) (Figure 4C). In the case of wild type strain Y51, a 5.0 kb fragment was detected in cells cultivated in the absence of CF, but in cells cultivated with CF the intensity of this 5.0 kb band was significantly reduced and a 3.5 kb band was detected (Figure 4B). The 3.5 kb fragment was derived from a PCE-nondechlorinating SD variant that emerged following deletion of the IS element located upstream of the *pceA* gene, as described previously (Figure 4C) (Futagami et al. 2006b).

In the case of strain TCE1, DNA bands of 9.4 kb and 6.0 kb were detected when the cells were cultivated in the absence of CF. The 9.4-kb DNA fragment was derived from chromosomal DNA containing *pceA*, whereas the 6.0-kb DNA fragment was derived from circular DNA containing *pceA*, which was generated by homologous recombination between the two identical IS elements, as shown in Figure 4C (Maillard et al. 2005). The intensity of these two *pce*-containing bands was significantly reduced when strain TCE1 was cultivated in the presence of CF, indicating that the *pce*-deletion mutant became dominant after the cells were cultivated in the presence of CF. Thus, the *pce* gene cluster is very unstable, and is deleted in the presence of CF in both strains Y51 and TCE1.

## Discussion

In this study, we investigated the effects of CF on the *ortho*-chlorophenol-dechlorinating bacteria *D. dehalogenans* strain JW/IU-1 and *D. hafniense* strain DCB-2. The growth of both strains was inhibited by CF at a concentration of 100 μM in medium containing 3-Cl-4-OHPA as the electron acceptor (Figure 1). It is noteworthy that 100 μM CF did not directly inhibit the dechlorination of 3-Cl-4-OHPA by resting cells (Figure 2). However, the dechlorination activity of cells cultivated in the presence of 100 μM CF was lower than that of cells cultivated in the absence of CF. This reduction in dechlorination activity was due to decreased transcription of *cprA* in the presence of CF (Figure 3). These results suggest that inhibition of growth mediated by CF in medium containing 3-Cl-4-OHPA is due to inhibition of CprA expression, as this protein is essential for organohalide respiration utilizing 3-Cl-4-OHPA.

In strains JW/IU-1 and DCB-2, transcription of the *cpr* genes is strictly regulated by CprK, which is a member of the cAMP-binding protein-fumarate nitrate reduction regulatory protein (CPR-FNR) family of transcriptional factors (Pop et al. 2004;Gabor et al. 2006). The interaction of a chlorinated aromatic substrate such as 3-Cl-4-OHPA with the effector domain of CprK triggers binding of CprK to a DNA sequence called the dehalobox. Binding of CprK to the dehalobox leads to transcriptional activation of the *cpr* gene cluster. Thus, CF might act as a competitive inhibitor of 3-Cl-4-OHPA, keeping CprK in an inactivated state.

The above-described mechanism through which CF inhibits the growth of *Desulfitobacterium* is supported by the observed differences in the degree that CF inhibited the growth of strain JW/IU-1 under different cultivation conditions. In the presence of 100 μM CF, growth rate of JW/IU-1 was inhibited in medium containing 3-Cl-4-OHPA, but not in medium containing fumarate as the electron acceptor, which may be explained by that the expression of CprA is not required for fumarate respiration. In fact, it was demonstrated that the transcriptional level of *cprA* in the medium containing fumarate as an electron acceptor was significantly lower than that in a medium containing 3-Cl-4-OHPA as an electron acceptor (Smidt et al. 2000). On the other hand, CF inhibited the growth of strain DCB-2 to a greater degree than strain JW/IU-1. Even at a concentration as low as 1 μM, CF inhibited the growth of strain DCB-2 in medium containing either 3-Cl-4-OHPA or fumarate. Moreover, CF inhibited the growth rate of strain JW/IU-1, whereas CF caused both the extended lag phase and the reduced cell yield of strain DCB-2. This result indicates that a direct toxic effect of CF on the overall fitness of the cells of strain DCB-2 also present. Similar inhibitory effects of organic solvents have been observed for anaerobic bacteria (Duldhardt et al. 2007;Duldhardt et al. 2010).

CF inhibited the growth of the PCE- and TCE-dechlorinator *D. hafniense* strain TCE1. This result was similar to that observed in our previous study involving *D. hafniense* strain Y51 (Futagami et al. 2006b). Southern blot analysis using *pceA* as a probe showed that the signal intensity associated with *pceA* decreased significantly when cells of strain TCE1 were cultivated in the presence of CF, indicating that the *pceA*-deletion variant became dominant. Although the *pce* genes of strains TCE1 and Y51 are very similar (>99%, including the intergenic regions) (Maillard et al. 2005;Futagami et al. 2006a) and CF had a similar inhibitory effect on the growth of both strains, one difference was the occurrence of the circular *Tn*-Dha1 DNA element containing the entire *pce* gene cluster (Figure 4B and C). The band derived from the *Tn*-Dha1 element formed by homologous recombination between the two identical IS elements was detected by Southern blot analysis, as described previously (Maillard et al. 2005). Strain Y51 also has the same type of circular element, designated Tn*Desp1* (Futagami et al. 2006a), with a predicted band size on Southern blotting of ca. 6.5 kb. However, no 6.5-kb DNA band was detected under the cultivation conditions used in this study, indicating that the efficiency of excision by homologous recombination between the IS elements and/or the stability of the circular elements of strains TCE1 and Y51 might be different.

In conclusion, CF had a negative effect on all the *Desulfitobacterium* strains tested, including *D. dehalogenans* strain JW/IU-1, *D. hafniense* strain DCB-2, and *D. hafniense* strain TCE1. Because the *Desulfitobacterium* play a significant role in microbial reductive dechlorination, the presence of CF in the environment could be detrimental to bioremediation efforts aimed at other halogenated hydrocarbons. The degradation of CF through organohalide respiration and fermentation by *Dehalobacter* spp. was recently reported (Grostern et al. 2010;Lee et al. 2012). The results of these studies could be important for developing effective strategies for the bioremediation of halogenated compounds in anaerobic environments.

## Competing interest

The authors declare that they have no competing interests.
